# Comparative effectiveness of lactulose and sennosides for the prevention of peritoneal dialysis-related peritonitis: an open-label, randomized, active-controlled trial

**DOI:** 10.1080/07853890.2021.1889023

**Published:** 2021-02-17

**Authors:** Kajohnsak Noppakun, Tichanun Narongchai, Romanee Chaiwarith, Uraiwan Wongsawad, Surachet Vongsanim, Chidchanok Ruengorn, Surapon Nochaiwong

**Affiliations:** a Division of Nephrology, Department of Internal Medicine, Faculty of Medicine, Chiang Mai University, Chiang Mai, Thailand; b Acute Dialysis Unit, Maharaj Nakorn Chiang Mai Hospital, Faculty of Medicine, Chiang Mai University, Chiang Mai, Thailand; c Pharmacoepidemiology and Statistics Research Center (PESRC), Faculty of Pharmacy, Chiang Mai University, Chiang Mai, Thailand; d Division of Infectious Disease and Tropical Medicine, Department of Internal Medicine, Faculty of Medicine, Chiang Mai University, Chiang Mai, Thailand; e Department of Pharmaceutical Care, Faculty of Pharmacy, Chiang Mai University, Thailand

**Keywords:** Constipation, lactulose, peritonitis, peritoneal dialysis, prevention, *Senna*

## Abstract

**Background:**

To the best of our knowledge, the effectiveness and safety of lactulose in comparison to sennosides, for the prevention of peritoneal dialysis (PD)-related peritonitis, has never been tested in a randomized study.

**Methods:**

We conducted an open-label, randomized, active-controlled trial in a PD-center in Northern Thailand. Adult patients on PD were enrolled and randomly assigned in a 1:1 ratio into two groups; one group received lactulose 15 mL once daily (*n* = 50) and the other group received sennosides two tablets daily (*n* = 50). The primary outcome was time-to-first bacterial peritonitis. The secondary outcomes included a composite of bacterial peritonitis and all-cause mortality. Cox proportional hazards regression was calculated and presented as hazard ratios (HRs) with 95% confidence intervals (CIs).

**Results:**

One hundred PD patients were recruited (50.0% men; mean age 55.5 ± 13.0 years) in this study. The baseline characteristics of the study participants were similar in both groups. No significant trend towards a higher risk of PD-related peritonitis was observed in the lactulose group (HR, 2.32 [95% CI, 0.92–5.83]; *p* = .051) compared to the sennosides group. Nevertheless, the secondary outcome was significantly higher in the lactulose group (HR, 2.77 [95% CI, 1.20–6.41]; *p* = .010). The incidence of adverse events was not substantially different between the two groups; however, diarrhoea was more frequent in the lactulose group (38.0% vs. 18.0%; *p* = .030) than in the sennosides group.

**Conclusions:**

Treatment with lactulose is not more effective than sennosides and cannot be routinely recommended for the prevention of peritonitis among the PD population.

**TRIAL REGISTRATION**
Thai Clinical Trial Registry (clinicaltrials.in.th); ID: TCTR20171012001

**KEY MESSAGE**
- To the best of our knowledge, no randomized controlled trial that compares the efficacy and safety profiles of lactulose versus sennosides for the prevention of PD-related peritonitis among the PD population has been conducted.
- In this open-label, randomized, active-controlled trial, treatment with lactulose is not more effective than sennosides in the prevention of PD-related peritonitis, and it could increase the risk of bacterial PD-related peritonitis.
- Further studies with a larger sample size by incorporated real-world evidence are needed to confirm our findings and to explore strategies to prevent peritonitis among PD patients.

## Introduction

Despite the improvement in innovations of peritoneal dialysis (PD) and treatment-care management over the past three decades, PD-related peritonitis remains a debilitating and serious complication among patients with end-stage kidney disease undergoing PD worldwide [1,2]. PD-related peritonitis has not only increased the risk of morbidity, hospitalization, and mortality, but is also a common leading cause of modality change, and consequently, is expensive to manage [2–4]. Theoretically, Gram-positive organisms, particularly common skin organisms, cause PD-related peritonitis through poor technique and contamination of the PD catheter and connection systems. Invasive endoscopic procedures (e.g. colonoscopy and gynaecologic procedures) are also an important route of PD-related peritonitis infection. Gram-negative organisms related to peritonitis, specifically enteric organisms, may contribute to the poor technique and transmural migration of organisms across the bowel wall [5].

In most circumstances, numerous controlled trials with antimicrobial approaches have attempted to decrease the risk of Gram-positive organism-related peritonitis, primarily *Staphylococcus aureus*; however, limited information is available regarding Gram-negative organisms, particularly enteric organisms. According to an international prospective study, the Peritoneal Dialysis Outcomes and Practice Patterns Study (PDOPPS), the microbiology of PD-related peritonitis was similar across various countries (Australia, New Zealand, Canada, Japan, the United Kingdom, and the United States; mainly Gram-positive organisms). However, the microbiology was different in Thailand, where Gram-negative and culture-negative organisms were more common [6]. Observational studies revealed that constipation, enteritis, and hypokalaemia are associated with an increased risk of PD-related peritonitis by enteric organisms, particularly Gram-negative *Enterobacteriaceae*, possibly *via* delayed gut transit or gastrointestinal dysmotility, leading to increased transmural migration of organisms [7,8].

Chronic constipation is common in PD patients, with prevalence ranging from 14.2% to 90.3% [9]. Evidence revealed that constipation is associated with a higher risk of PD-related peritonitis [10]. As such, prevention of constipation using laxatives may be a reasonable strategy to prevent peritonitis in PD patients. In clinical practice, two types of laxatives are commonly used, specifically osmotic laxatives and stimulants. Lactulose, a non-absorbable disaccharide, is an osmotic laxative that is metabolized by bacteria in the colon into low molecular weight acids, mainly lactic and acetic acids, which can produce marked acidification of the colonic milieu [11]. Along with its osmotic effects, the acidification effects of lactulose can decrease bacterial colonization and increase colonic motility, which can decrease colonic bacterial concentration. In addition, fermentation of lactulose by colonic bacteria can produce short-chain fatty acids that stimulate the proliferation of intestinal mucosa, which can strengthen the mucosal barrier [12]. An observational study has shown that the regular use of lactulose is associated with a lower incidence of peritonitis in PD patients [13]. Sennosides are stimulant laxatives derived from the plants belonging to the genus *Senna*. Sennosides work by altering electrolyte transport in the colonic mucosa, increasing intraluminal fluids, and acting as irritants on the mucosa, and consequently, increase colonic movement [14]. Sennosides are effective laxatives; however, physicians still do not use them frequently, since no evidence regarding their effectiveness from well-designed randomized controlled trials is available. Further, the long-term safety of sennosides is still debated [15].

To date, data on preventing peritonitis in PD patients are scarce with a few high-quality randomized controlled trials. Consensus regarding the optimal strategy for the prevention of constipation has not been reached, and only a few prospective controlled studies on the use of laxative agents in PD patients have been published. To the best of our knowledge, no controlled study to compare the efficacy and detrimental impact of lactulose and sennosides in PD patients has been performed. In this lactulose and sennosides for the prevention of PD-related peritonitis (LACSOPP) trial, we aimed to assess the efficacy and safety profiles of lactulose and sennosides in the prevention of PD-related peritonitis.

## Methods

### Study design

The LACSOPP is an active-controlled, open-label, single-center randomized controlled trial. The trial was conducted at the Maharaj Nakorn Chiang Mai Hospital, Chiang Mai University, University Hospital of Northern Thailand between June 2017 and May 2018. The study protocol was approved by the Institutional Review Board of the Faculty of Medicine, Chiang Mai University, and has been registered with the Thai Clinical Trials Registry (clinicaltrials.in.th; study number: TCTR20171012001). The study was conducted in accordance with the 1964 Declaration of Helsinki and its amendments or comparable ethical standards. Written informed consent was obtained from all the participants in this study. The trial was reported according to the Consolidated Standards of Reporting Trials (CONSORT) Statement [16].

### Study population

Participants were enrolled in this study as per the following inclusion criteria: (i) patients aged 18 years or older at the date of screening; (ii) those who understood the trial protocol and were willing to provide written informed consent; and (iii) those with end-stage kidney disease who had been undergoing PD for at least 2 weeks. The exclusion criteria were as follows: (i) current or recent diarrhoea (within 1 week) before the date of screening; (ii) history of PD-related peritonitis or exit-site and tunnel infections within 3 months before the date of screening; (iii) a history of organ transplantation or plan to undergo organ transplantation or transfer to other centres within 6 months; (iv) pregnancy or plan to conceive; (v) contraindication or a history of allergy to lactulose or sennosides; (vi) currently participating in other studies; (vii) had any conditions (both mental or physical) that would interfere with participants’ ability to comply with the trial protocol; and (viii) prognosis for survival of less than 12 months or ongoing clinically significant illness.

### Randomization

Eligible participants were randomized with a 1:1 allocation ratio into two groups to receive lactulose or sennosides. Randomisation using randomly permuted blocks stratified according to the duration of PD treatment (less than or more than 1 year) and history of PD-related peritonitis was performed by the study coordinators who were not involved with the practice management care. The randomisation assignment was concealed until all eligibility criteria had been met. Blinding was not maintained owing to the nature of the intervention. After the investigators had obtained the participants’ consent, a person independent of the recruitment process was contacted through telephone for allocation assignment.

### Intervention and study procedures

Participants were randomly assigned to receive either lactulose (Berlin Pharm, Thailand) or sennosides (7.5 mg total sennosides per tablet; SPS Medical, Thailand). The dosages of both laxatives were adjusted to keep the frequency of bowel movements between twice a day and once every 2 days. Participants were instructed to contact investigators to adjust the laxative dosage if they had bowel movement beyond the target range.

The dosage was initiated at 15 mL once daily, and was then titrated by an increase of 15 mL every 3 days to a maximum of 45 mL once daily for the lactulose group. If participants developed diarrhoea, the dosage was decreased by 5 mL daily. If diarrhoea persisted with a dosage of 5 mL daily, lactulose was discontinued. Furthermore, for the sennosides group, the dosage was started at two tablets once daily, then increased by one tablet every 3 days to a maximum of four tablets daily. If participants developed diarrhoea, the dose was decreased by one tablet every day and was discontinued if the participants still had diarrhoea at a dose of one tablet daily.

All the participants in this trial were administered intravenous antibiotics at the time of PD catheter insertion, using the Y-set and double-bag systems, and using conventional glucose-based solutions. Routine exit-site care with prophylactic antimicrobial agents was not adopted in our PD setting owing to concerns regarding the emergence of resistance to antimicrobial agents. However, all the participants and their caregivers in this trial were trained with a standard program for daily exit-site care with normal saline dressing along with sterile techniques focussing on hand hygiene and mask-wearing during PD bag exchange. Adherence to interventions and concomitant medications as a part of standard PD treatment was recorded and monitored by direct observation of the participants and their caregivers, and by counting the quantity of assigned medication on each follow-up visit. Treatment adherence was defined as more than 80% of the participants taking the assigned medication.

### Assessment of outcomes and adverse events

Participants were assessed and followed-up monthly for at least 6 months from the baseline along with routine outpatient PD clinic visits and were censored at 1 year after randomization or at the end of the study, whichever came first. The baseline sociodemographic data, PD treatment characteristics, clinical characteristics, and routine laboratory test results were documented each follow-up visit. All components of the outcomes were reviewed and adjudicated by the assigned investigator (K.N.) who was unaware of the treatment assignment.

The primary outcome was the time-to-first bacterial PD-related peritonitis as defined by the 2016 International Society for Peritoneal Dialysis (ISPD) guidelines [3]. Participants were diagnosed with PD-related peritonitis if they had at least two of the following criteria: (i) abdominal pain and/or cloudy dialysis effluent; (ii) dialysis effluent with white blood cell count more than100 cells/mm^3^ (after a dwell time of at least 2 h), with polymorphonuclear leukocytes 50% or more; and (iii) positive effluent Gram stain and/or culture. In patients in whom peritonitis was suspected, 2 L of dialysate was instilled and drained after 2 h to check for cell count and culture. Cases of culture-negative PD-related peritonitis, which were treated with a combination of intraperitoneal antibiotics (cefazolin and ceftazidime) for 2 weeks, were also defined as bacterial peritonitis [17]. The secondary outcome was the composite outcome of bacterial PD-related peritonitis and death from any cause.

Safety profiles in terms of any adverse events (both serious and non-serious) were documented in detail. Adverse events were spontaneously reported or recorded after an inquiry using an open-ended question. A serious adverse event was defined as an adverse event related to all-cause mortality, life-threatening illness, disability, hospital admission, or prolongation of hospital stay. Furthermore, stool consistency was closely monitored using the Bristol stool scale, which has been validated for clinical research [18]. Diarrhoea was defined as three or more bowel movements per day in combination with stool consistency compatible with the Bristol stool scale type 5–7; constipation was defined as one or less bowel movement in 3 days in combination with the Bristol stool scale types 1 and 2 [19]. All diarrhoea and constipation events were evaluated after a stable dose of laxatives was administered to each patient for at least 1 week. However, diarrhoea is one of the manifestations of PD-related peritonitis and can present in up to a quarter of patients with *Enterobacteriaceae* PD-related peritonitis [20]. Any episode of diarrhoea reported within 3 days before the date of PD-related peritonitis diagnosis was not considered an adverse event.

### Statistical analyses

We calculated the sample size based on a previous report, which stated that the annual rate of PD-related peritonitis in the sennosides and lactulose groups was 50% and 20%, respectively [13]. On the basis of the log-rank test of survival functions to obtain 80% power with a two-sided significance level of 5% and an all-cause dropout rate of 15%, approximately 45 participants were required in each group.

Baseline characteristics are expressed as number (percentage) or mean ± standard deviation (SD), or medians with interquartile range (IQR) as appropriate. Differences between groups were compared using the Chi-square test or Fisher’s exact test for categorical data and independent t-test or Wilcoxon rank-sum test for continuous data. All analyses for efficacy and safety were based on the intention-to-treat principle. Cox proportional hazards regression and treatment effects were calculated and estimated as hazard ratios (HRs) with corresponding 95% confidence intervals (CIs) for the time-to-event outcomes. The Kaplan–Meier survival curves were generated to visualize the time-to-event comparisons. The proportional hazard assumption was tested by the plots of Schoenfeld residual, and no violation was observed. A repeated measure mixed-effects model to test the difference between groups was analyzed for continuous variables measured during the trial follow-up.

Preplanned subgroup analyses were performed based on the participant characteristics, including age (<65 vs. ≥65 years), sex, history of diabetes, duration of PD treatment (<1 vs. ≥1 year), PD modality (continuous ambulatory PD [CAPD] vs. automated PD [APD]), history of PD-related peritonitis, history of laxative use, and serum albumin (<3.5 vs. ≥3.5 mg/dL), and potassium (<3.5 vs. ≥3.5 mmol/L) levels. All tests were two-sided, with a *p*-value of less than .05 indicating statistical significance. All statistical analyses were performed using the Stata software version 15.0 (StataCorp, LP).

## Results

### Study population

**Figure 1.**
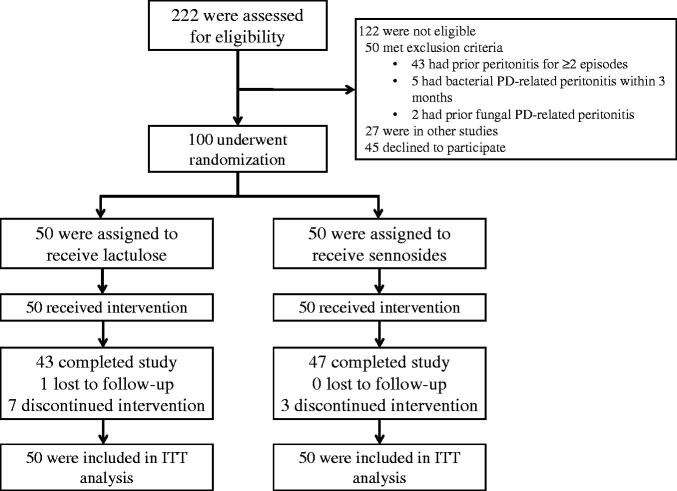
Study flow diagram. Abbreviations. ITT: intention-to-treat; PD: peritoneal dialysis.

A total of 222 PD patients were screened. Of these, 100 patients were eligible for the study and underwent randomization (50 patients in each group) (Figure 1). The baseline characteristics were well balanced between the two groups (Table 1). The mean age of the patients included in this study was 55.5 ± 13.0 years; 50% of the patients were men, and 34% had a history of diabetes mellitus. The median PD vintage was 34.0 months (IQR, 17.0–54.2), and 30% had a history of PD-related peritonitis. Laxatives were regularly used by 55% of the patients before enrolment; all of these patients were administered sennosides. According to the Bristol stool scale, 98% of the patients had normal stool consistency (Bristol stool scale types 3 and 4), while only 2% had constipation at baseline.

**Table 1.** Baseline characteristics of PD patients in the LACSOPP trial.

Characteristics	Total (*n* = 100)	Lactulose (*n* = 50)	Sennosides (*n* = 50)	*p*-Value
Age, year	55.5 ± 13.0	57.0 ± 12.8	54.1 ± 13.1	.262
Male	50 (50.0)	25 (50.0)	25 (50.0)	1.000
Body weight, kg	57.4 ± 12.5	56.2 ± 12.2	58.6 ± 12.7	.335
Diabetes mellitus	35 (35.0)	18 (36.0)	17 (34.0)	1.000
Duration of PD, year				
<1	19 (19.0)	9 (18.0)	10 (20.0)	.624
≥1	81 (81.0)	41 (82.0)	40 (80.0)	
PD modality				
APD	13 (13.0)	6 (12.0)	7 (14.0)	1.000
CAPD	87 (87.0)	44 (88.0)	43 (86)	
History of PD-related peritonitis	30 (30.0)	15 (30.0)	15 (30.0)	1.000
Laxative use before randomisation	55 (55.0)	29 (58.0)	26 (52.0)	.688
Bristol stool scale				
Type 1–2	2 (2.0)	2 (4.0)	0 (0.0)	.959
Type 3–4	98 (98.0)	48 (96.0)	50 (100.0)	
Urine output, ml per day				
<100	31 (31.0)	16 (32.0)	15 (30.0)	.937
100–400	33 (33.0)	17 (34.0)	16 (32.0)	
>400	36 (36.0)	17 (34.0)	19 (38.0)	
Blood urea nitrogen, mg/dL	47.8 ± 13.8	48.5 ± 13.1	47.2 ± 14.5	.619
Serum creatinine, mg/dL	9.6 ± 3.4	9.2 ± 3.0	9.9 ± 3.9	.307
Serum sodium, mmol/L	138.9 ± 3.7	139.0 ± 3.8	138.8 ± 3.6	.766
Serum potassium, mmol/L	4.1 ± 0.7	4.1 ± 0.8	4.1 ± 0.7	.756
Serum bicarbonate, mmol/L	26.8 ± 2.7	27.1 ± 2.7	26.5 ± 2.6	.280
Serum calcium, mg/dL	8.8 ± 1.0	8.7 ± 1.1	8.9 ± 1.0	.292
Serum phosphate, mg/dL	4.3 ± 1.3	4.2 ± 1.2	4.5 ± 1.4	.260
Blood glucose, mg/dL	109.7 ± 39.5	108.0 ± 31.5	111.3 ± 46.4	.682
Serum albumin, g/dL	3.7 ± 0.5	3.6 ± 0.4	3.7 ± 0.6	.601

Data are presented as mean ± SD or number (%).

Abbreviations. APD: automated peritoneal dialysis; CAPD: continuous peritoneal dialysis; PD: peritoneal dialysis; LACSOPP: LACtulose and Sennosides or the preventiOn of Peritoneal dialysis-related Peritonitis.

The median follow-up time in lactulose and sennosides groups was 9.4 (IQR, 6.1–9.8) and 9.7 months (IQR, 9.3–10.2), respectively, and the median follow-up time of this trial was 9.5 months (IQR, 7.7–10.0). Only one patient was lost to follow-up, and 10 patients (10%; seven in the lactulose group and three in the sennosides group) prematurely discontinued the study medications (Figure 1). Diarrhoea was the main reason for discontinuation of the medications. The median dose of lactulose was 10 mL daily (range, 0–15), and the median dose of sennosides was two tablets daily (range, 0–3).

### Efficacy endpoints

**Figure 2.**
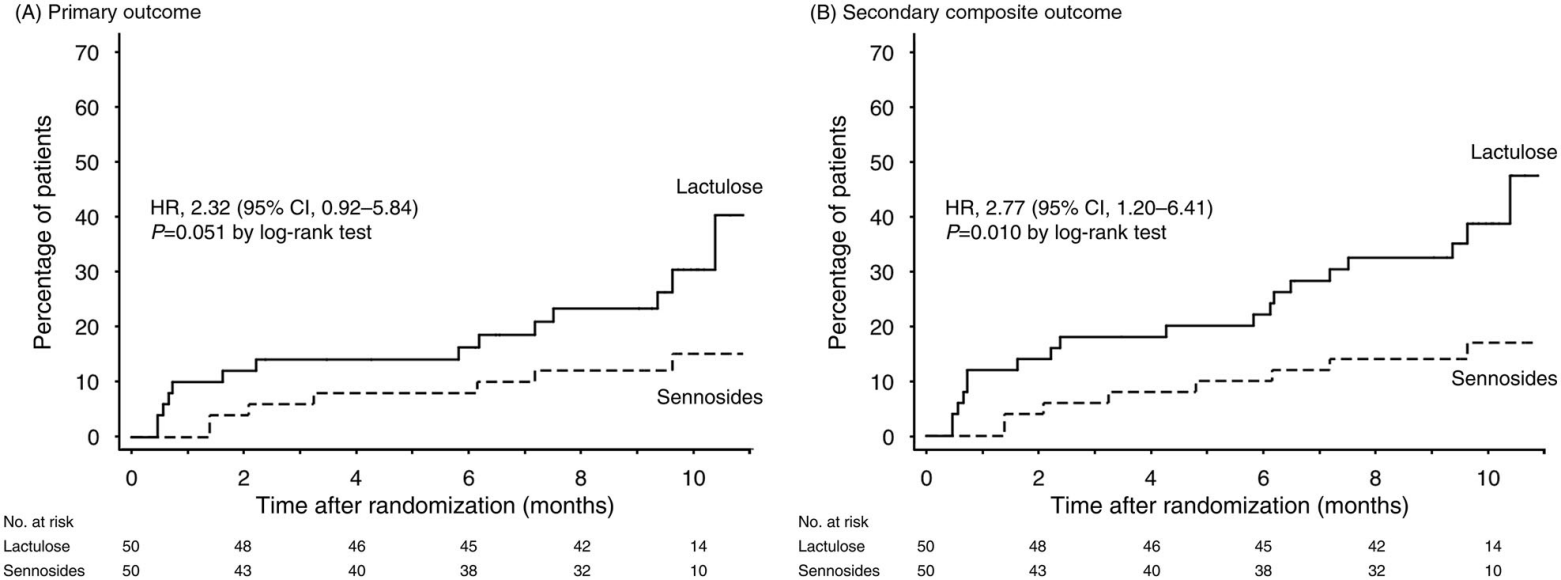
Kaplan–Meier plot of primary and secondary outcomes. (A) Time-to-first Bacterial PD-related peritonitis. (B) Secondary composite outcome of bacterial PD-related peritonitis and death from any cause. Abbreviations. CI: confidence interval; HR: hazard ratio; PD: peritoneal dialysis.

Of the total study participants, 21 (21.0%) participants developed PD-related peritonitis, which was the primary outcome. During the study period, the incidence rates of bacterial peritonitis were 0.45 (95% CI, 0.26–0.75) and 0.19 (95% CI, 0.09–0.39) episodes per patient-year in the lactulose and sennosides groups, respectively (*p* = .058), with no significant trend towards shorter time to develop bacterial PD-related peritonitis in the lactulose group (HR, 2.32; 95% CI, 0.92–5.84; *p* = .051; Figure 2(A)). Of the 21 patients who developed bacterial PD-related peritonitis (Table 2), the most common organism isolated was group D streptococci (19.0%), followed by *Escherichia coli* (14.3%) and *Klebsiella pneumoniae* (14.3%). Culture-negative PD-related peritonitis was detected in 28.6% of patients. No difference between the two groups with regard to the organisms isolated from the peritoneal fluid cultures was observed (*p* = .480).

**Table 2.** Number and percentage of organisms causing PD-related peritonitis.

Organisms	Lactulose (*n* = 14)	Sennosides (*n* = 7)	Total (*n* = 21)
Group D streptococci	1 (7.1)	3 (42.8)	4 (19.0)
*Staphylococcus aureus*	1 (7.1)	0 (0.0)	1 (4.8)
*Escherichia coli*	3 (21.4)	0 (0.0)	3 (14.3)
*Klebsiella pneumoniae*	2 (14.3)	1 (14.3)	3 (14.3)
*Acinetobacter baumannii*	1 (7.1)	0 (0.0)	1 (4.8)
*Neisseria spp.*	1 (7.1)	0 (0.0)	1 (4.8)
*Micrococcus spp.*	1 (7.1)	0 (0.0)	1 (4.8)
Non-fermenting gram-negative bacilli	0 (0.0)	1 (14.3)	1 (4.8)
Negative culture	4 (28.6)	2 (28.6)	6 (28.6)

Data are presented as number (%). Percentages may not total 100 because of rounding.

Abbreviation. PD: peritoneal dialysis.

Nineteen patients (38.0%) in the lactulose group and nine patients (18.0%) in the sennosides group developed the secondary outcome of the composite of bacterial PD-related peritonitis and death from any cause (HR, 2.77; 95% CI, 1.20–6.41; *p* = .010; Figure 2(B)). Nine patients (9.0%) died during the study period, in which four patients died from sepsis, three from unknown causes, presumably sudden cardiac death, and one each from acute myocardial infarction and gastrointestinal haemorrhage. Five patients (0.16 episodes per patient-year; 95% CI, 0.07–0.38) in the lactulose group and one patient (0.027 episodes per patient-year; 95% CI, 0.004–0.190) in the sennosides group developed *Enterobacteriaceae* PD-related peritonitis (*p* = .080). Body weight and serum chemistry results during the follow-up visits did not differ between the two groups (Supplementary Figure S1). According to the set of subgroup analyses, no significant difference was found between the lactulose and sennosides groups (all *p* for interaction >.05, Supplementary Figure S2).

### Adverse events

All randomized patients (*n* = 100) were included in the analysis of adverse events. No difference in adverse events and serious adverse events between the two groups (Table 3). Diarrhoea was more common in the lactulose group (38.0% vs. 18.0%; *p* = .044) than in the sennosides group. Exit-site infection, hyperkalemia, hypertension, and upper respiratory tract infection were more common in the sennosides group although not statistically significant. The incidence of other adverse events, including constipation and hypokalaemia, was similar in both the groups.

**Table 3.** Adverse events occurring in patients received lactulose and sennosides.

Event	Lactulose (*n* = 50)	Sennosides (*n* = 50)	*p*-Value
All adverse events	47 (94.0))	48 (96.0)	.650
Serious adverse events	7 (14.0)	12 (24.0)	.200
Diarrhoea	19 (38.0)	9 (18.0)	.044
Constipation	1 (2.0)	1 (2.0)	1.000
Dyspepsia	2 (4.0)	2 (4.0)	1.000
Exit site infection	7 (14.0)	11 (22.0)	.300
PD catheter removal	2 (4.0)	1 (2.0)	1.000
Hyponatremia (serum sodium <135 mmol/L)	2 (4.0)	1 (2.0)	1.000
Hypokalaemia (serum potassium <3.5 mmol/L)	32 (64.0)	32 (64.0)	1.000
Hyperkalemia (serum potassium ≥5.5 mmol/L)	1 (2.0)	5 (10.0)	.204
Acidosis (serum bicarbonate <22 mmol/L)	1 (2.0)	2 (4.0)	1.000
Hyperglycemia	4 (8.0)	3 (6.0)	1.000
Hyperthyroidism	1 (2.0)	0 (0.0)	1.000
Myelodysplastic syndrome	1 (2.0)	0 (0.0)	1.000
Iron deficiency anaemia	1 (2.0)	1 (2.0)	1.000
Heart failure	2 (4.0)	3 (6.0)	1.000
Volume overload	3 (6.0)	2 (4.0)	1.000
Atrial fibrillation	1 (2.0)	1 (2.0)	1.000
Hypertension (B*p* ≥ 140/90 mmHg)	8 (16.0)	12 (24.0)	.454
Hypotension (B*p* ≤ 90/60 mmHg)	4 (8.0)	3 (6.0)	1.000
Syncope	0 (0.0)	2 (4.0)	.495
Dizziness	4 (8.0)	6 (12.0)	.741
Seizure	1 (2.0)	2 (4.0)	1.000
Upper respiratory tract infection	1 (2.0)	5 (10.0)	.204
Pneumonia	1 (2.0)	0 (0.0)	1.000
Myalgia	3 (6.0)	1 (2.0)	.617
Cellulitis	0 (0.0)	1 (2.0)	1.000
Osteomyelitis	1 (2.0)	0 (0.0)	1.000
Septic arthritis	1 (2.0)	0 (0.0)	1.000
Gouty arthritis	0 (0.0)	1 (2.0)	1.000
Back abscess	1 (2.0)	0 (0.0)	1.000

Data are presented as number (%).

Abbreviation. PD: peritoneal dialysis.

## Discussion

We conducted this study as, to the best of our knowledge, no randomized controlled trial that compares the efficacy and safety profiles of lactulose versus sennosides for the prevention of PD-related peritonitis among the PD population has been conducted. A 2.3-fold increase with a borderline statistical significance for the incidence of bacterial PD-related peritonitis was noted in the lactulose group. With respect to the secondary outcomes, the study showed that the composite of bacterial PD-related peritonitis and death from any cause was significantly higher in the lactulose group; however, death from any cause alone was not different between the two groups. The incidence of *Enterobacteriaceae* PD-related peritonitis was marginally higher in those receiving lactulose. *Escherichia coli* and *K. pneumoniae* were the most common Gram-negative organisms among these PD patients who had positive culture results.

The results of this study contradict those of previous observational study, which showed that the risk of developing PD-related peritonitis was lowered by one-fifth in patients who regularly use lactulose (HR, 0.23; 95% CI, 0.06–0.80; *p* = .021), and that the incidence of Gram-negative PD-related peritonitis was lower in patients receiving lactulose [13]. The use of lactulose to prevent PD-related peritonitis is derived from the evidence that lactulose can hamper bacterial translocation from the intestinal lumen into the peritoneal cavity [21]. However, data supporting this concept are controversial. Studies from an animal model have shown that colonic bacteria, specifically *Enterobacteriaceae*, may undergo transmural migration into the peritoneal cavity if colonic mucosa is inflamed, and lactulose can significantly reduce bacterial translocation [22,23]. Nevertheless, other studies have shown that even though lactulose can inhibit intestinal bacterial colonization, it does not improve and may promote bacterial translocation in an animal model [24,25].

Both constipation and diarrhoea have been recognized as risk factors for PD-related peritonitis [10]. In addition, a case series of five patients showed that acute treatment of constipation could increase the risk of PD-related peritonitis [26]. The relationship between constipation, diarrhoea, and an episode of PD-related peritonitis can explain the contradictory results of this study. Furthermore, this relationship can also explain the results of the previous study in which none of the 17 patients in the lactulose group developed diarrhoea despite receiving large doses of lactulose, starting at 30 mL once daily to 30 mL twice daily [13]. By contrast, the median dose of lactulose in our study was only 10 mL, with a maximum of 15 mL daily. Even with this remarkably lower lactulose dosage, 38% of our patients who received lactulose still developed diarrhoea. Additionally, a previous study showed that 23.5% of patients in the lactulose group and 45.6% of patients in the non-lactulose group developed constipation [13], although this difference was not statistically significant; the two-time difference certainly has an impact on the incidence of PD-related peritonitis. Meanwhile, only 1% of patients in the lactulose or sennosides group in our study developed constipation. The differences in the rate of diarrhoea and constipation between the lactulose and sennosides groups in our study and the previous study may explain the higher incidence of bacterial PD-related peritonitis and *Enterobacteriaceae* PD-related peritonitis in the lactulose group in our study.

With regard to the significant increase in the incidence of the composite secondary outcome of bacterial PD-related peritonitis and death from any cause in the lactulose group, we speculated that there could be a common risk factor for these two conditions in the patients receiving lactulose. Hypokalaemia has been proposed as a risk factor for *Enterobacteriaceae* PD-related peritonitis [7]. Several mechanisms have been postulated regarding the risk factors, including hypokalaemia, which may lead to decreased bowel movements [26], constipation, and consequently, bacterial overgrowth [8]. Furthermore, hypokalaemia is closely associated with malnutrition, which could lead to oedema of the bowel wall and impaired immune function [7,27]. Hypokalaemia and malnutrition can increase the risk of PD-related peritonitis, as well as mortality [28]. The findings from the study did not confirm our hypothesis because the proportion of patients with hypokalaemia at the baseline and during the follow-up period was similar in both the groups. Furthermore, body weight, blood urea nitrogen, and serum albumin levels did not differ between the lactulose and sennosides groups. However, as the composite endpoint is not the primary outcome, the results need to be interpreted with caution, and further studies are required to confirm these findings. However, sennosides may increase intraluminal pressures, which raises concerns regarding the risk of bacterial PD-related peritonitis [29]. We found that sennosides were not inferior to lactulose in the prevention of PD-related peritonitis.

The strength of this study is the randomized controlled design, the use of a validated tool to assess bowel movement, high treatment regimen adherence rate (80.0%), and a low attrition rate (1%). In addition, all analyses performed in the study were based on the intention-to-treat principle; thus, selection bias could be minimized. However, this study had some limitations. First, this study was an open-label, as opposed to double-blind, design that could create an ascertainment bias, a limitation that may introduce bias through unblinding. However, we attempted to limit this bias using standardized criteria for the diagnosis of PD-related peritonitis; additionally, the investigator who assessed the outcomes was unaware of the treatment assignment. Second, an assumption regarding a 50% rate of PD-related peritonitis in the sennosides group was remarkably erroneous since only 14% of patients in the sennoside group experienced PD-related peritonitis. This assumption was based on data from an observational study of 57 patients who did not receive lactulose, which did not provide any information regarding the number of patients that received sennosides. Lastly, this study has insufficient statistical power to detect the differences in PD-related peritonitis rates and adverse events between the two groups. Further studies with a larger sample size and accurate postulation of the PD-related peritonitis rate in a control group could resolve these issues.

In conclusion, our findings revealed that lactulose is not more effective than sennosides in the prevention of PD-related peritonitis among the PD population, and it could increase the risk of bacterial PD-related peritonitis. Diarrhoea is more common in patients receiving lactulose. This study had a small size, lower than expected outcome of interest, and a short follow-up period; hence, future studies are needed that yield useful insights to explore the associations between laxative-induced diarrhoea and the development of PD-related peritonitis.

## Supplementary Material

Supplemental Material

## Data Availability

The steering committee of the LACSOPP trial will consider reasonable requests for the sharing of data. Requests should be made to the corresponding author.

## References

[1] Mehrotra R, Devuyst O, Davies SJ, et al. The current state of peritoneal dialysis. J Am Soc Nephrol. 2016;27(11):3238–3252. 27339663 10.1681/ASN.2016010112 PMC5084899

[2] Nochaiwong S, Ruengorn C, Koyratkoson K, et al. A clinical risk prediction tool for peritonitis-associated treatment failure in peritoneal dialysis patients. Sci Rep. 2018;8(1):14797. 30287920 10.1038/s41598-018-33196-2 PMC6172229

[3] Li PK, Szeto CC, Piraino B, et al. ISPD peritonitis recommendations: 2016 update on prevention and treatment. Perit Dial Int. 2016;36(5):481–508. 27282851 10.3747/pdi.2016.00078 PMC5033625

[4] Cho Y, Johnson DW. Peritoneal dialysis-related peritonitis: towards improving evidence, practices, and outcomes. Am J Kidney Dis. 2014;64(2):278–289. 24751170 10.1053/j.ajkd.2014.02.025

[5] Piraino B. Innovations in treatment delivery, risk of peritonitis, and patient retention on peritoneal dialysis. Semin Dial. 2017;30(2):158–163. 28066919 10.1111/sdi.12571

[6] Perl J, Fuller DS, Bieber BA, et al. Peritoneal dialysis-related infection rates and outcomes: results from the Peritoneal Dialysis Outcomes and Practice Patterns Study (PDOPPS). Am J Kidney Dis. 2020;76(1):42–53. 31932094 10.1053/j.ajkd.2019.09.016

[7] Chuang YW, Shu KH, Yu TM, et al. Hypokalaemia: an independent risk factor of Enterobacteriaceae peritonitis in CAPD patients. Nephrol Dial Transplant. 2009;24(5):1603–1608. 19103738 10.1093/ndt/gfn709

[8] Shu KH, Chang CS, Chuang YW, et al. Intestinal bacterial overgrowth in CAPD patients with hypokalaemia. Nephrol Dial Transplant. 2008;24(4):1289–1292. 18997162 10.1093/ndt/gfn617

[9] Zuvela J, Trimingham C, Le Leu R, et al. Gastrointestinal symptoms in patients receiving dialysis: a systematic review. Nephrology. 2018;23(8):718–727. 29468835 10.1111/nep.13243

[10] Su CY, Pei J, Lu XH, et al. Gastrointestinal symptoms predict peritonitis rates in CAPD patients. Clin Nephrol. 2012;77(04):267–274. 22445469 10.5414/cn107249

[11] Bown RL, Gibson JA, Sladen GE, et al. Effects of lactulose and other laxatives on ileal and colonic pH as measured by a radiotelemetry device. Gut. 1974;15(12):999–1004. 4448417 10.1136/gut.15.12.999 PMC1413067

[12] Scheppach W, Bartram P, Richter A, et al. Effect of short-chain fatty acids on the human colonic mucosa in vitro. JPEN J Parenter Enteral Nutr. 1992;16(1):43–48. 1738218 10.1177/014860719201600143

[13] Afsar B, Elsurer R, Bilgic A, et al. Regular lactulose use is associated with lower peritonitis rates: an observational study. Perit Dial Int. 2010;30(2):243–246. 20200371 10.3747/pdi.2009.00035

[14] Izzy M, Malieckal A, Little E, et al. Review of efficacy and safety of laxatives use in geriatrics. World J Gastrointest Pharmacol Ther. 2016;7(2):334–342. 27158549 10.4292/wjgpt.v7.i2.334 PMC4848256

[15] Leung L, Riutta T, Kotecha J, et al. Chronic constipation: an evidence-based review. J Am Board Fam Med. 2011;24(4):436–451. 21737769 10.3122/jabfm.2011.04.100272

[16] Schulz KF, Altman DG, Moher D, CONSORT 2010 statement: updated guidelines for reporting parallel group randomised trials. BMJ. 2010;340:c332. 20332509 10.1136/bmj.c332 PMC2844940

[17] Salzer WL. Peritoneal dialysis-related peritonitis: challenges and solutions. Int J Nephrol Renovasc Dis. 2018;11:173–186. 29928142 10.2147/IJNRD.S123618 PMC6001843

[18] Riegler G, Esposito I. Bristol scale stool form. A still valid help in medical practice and clinical research. Tech Coloproctol. 2001;5(3):163–164. 11875684 10.1007/s101510100019

[19] Longstreth GF, Thompson WG, Chey WD, et al. Functional bowel disorders. Gastroenterology. 2006;130(5):1480–1491. 16678561 10.1053/j.gastro.2005.11.061

[20] Szeto CC, Chow VC, Chow KM, et al. Enterobacteriaceae peritonitis complicating peritoneal dialysis: a review of 210 consecutive cases. Kidney Int. 2006;69(7):1245–1252. 16467787 10.1038/sj.ki.5000037

[21] Lupton JR, Coder DM, Jacobs LR. Long-term effects of fermentable fibers on rat colonic pH and epithelial cell cycle. J Nutr. 1988;118(7):840–845. 2839648 10.1093/jn/118.7.840

[22] Schweinburg FB, Seligman AM, Fine J. Transmural migration of intestinal bacteria; a study based on the use of radioactive *Escherichia coli*. N Engl J Med. 1950;242(19):747–751. 15412704 10.1056/NEJM195005112421903

[23] Ozaslan C, Turkcapar AG, Kesenci M, et al. Effect of lactulose on bacterial translocation. Eur J Surg. 1997;163(6):463–467. 9231859

[24] Sukhotnik I, Srugo I, Mogilner JG, et al. Effect of lactulose on bacterial translocation and intestinal adaptation in a rat model of short bowel syndrome. J Pediatr Gastroenterol Nutr. 2008;46(5):507–513. 18493204 10.1097/MPG.0b013e31815faa88

[25] Bovee-Oudenhoven IM, ten Bruggencate SJ, Lettink-Wissink ML, et al. Dietary fructo-oligosaccharides and lactulose inhibit intestinal colonisation but stimulate translocation of salmonella in rats. Gut. 2003;52(11):1572–1578. 14570725 10.1136/gut.52.11.1572 PMC1773861

[26] Singharetnam W, Holley JL. Acute treatment of constipation may lead to transmural migration of bacteria resulting in gram-negative, polymicrobial, or fungal peritonitis. Perit Dial Int. 1996;16(4):423–425. 8863339

[27] Noppakun K, Kasemset T, Wongsawad U, et al. Changes in serum albumin concentrations during transition to dialysis and subsequent risk of peritonitis after peritoneal dialysis initiation: a retrospective cohort study. J Nephrol. 2020;33(6):1275–1287. 32130719 10.1007/s40620-020-00716-1

[28] Vavruk AM, Martins C, Nascimento MM, et al. Association between hypokalemia, malnutrition and mortality in peritoneal dialysis patients. J Bras Nefrol. 2012;34(4):349–354. 23318823 10.5935/0101-2800.20120024

[29] Setyapranata S, Holt SG. The gut in older patients on peritoneal dialysis. Perit Dial Int. 2015;35(6):650–654. 26702007 10.3747/pdi.2014.00341 PMC4689468

